# A Rare Case of Domperidone-Induced Acute Dystonia in a Young Adult Due to Consumption of Combination Drug (Proton Pump Inhibitors With Domperidone) and Its Possible Pathomechanism

**DOI:** 10.7759/cureus.23723

**Published:** 2022-04-01

**Authors:** Bob Daripa, Scott Lucchese

**Affiliations:** 1 Internal Medicine/Neurology, Singapore General Hospital, Singapore, SGP; 2 Medicine, Grant Medical College and Sir J.J. (Jamsetjee Jeejeebhoy) Group of Government Hospitals, Mumbai, IND; 3 Neurology/Headache, University of Arkansas for Medical Sciences (UAMS), Little Rock, USA; 4 Neurology/Headache, University of Missouri School of Medicine, Columbia, USA

**Keywords:** dopamine receptor super sensitivity, extra pyramidal syndrome, acute dystonia, proton pump inhibitors (ppis), drug induced dystonia (did), domperidone, d2 receptor antagonist

## Abstract

Globally, a substantial number of people are tormented by dystonia. Domperidone, a D-2 receptor antagonist acts outside the blood-brain barrier in the brain stem as well as on the gastrointestinal tract. In India, domperidone is conveniently obtainable over the counter as a combination drug with proton pump inhibitors (PPIs) for dyspepsia and gastro-esophageal reflux disease. We present a rare case of domperidone-induced acute dystonia in a young adult presented within 72 hours after consuming two oral doses of this combination drug (PPIs with domperidone) for dyspepsia. Drug-induced extra pyramidal symptoms (EPS) are often misdiagnosed as some psychiatric condition and undoubtedly its expeditious diagnosis staves off unnecessary investigations and ameliorates prognosis. Our case ignites alertness amongst practitioners in India over the judicious use of PPIs with domperidone as the latter may trigger EPS. Such combination drugs can be prescribed if absolutely mandatory by the treating physician. The possible pathomechanism of this hyperkinetic motor phenomenon, perturbing the equilibrium of the cortical-subcortical circuit and resulting in an overflow of muscular activity, is attempted to be explained here, although the explicit mechanism is still blurry.

## Introduction

Oppenheime first delineated and coined the word ‘Dystonia’ in 1911 [1]. Today, we have a sizable amount of dystonic cases around the world. Domperidone, a D-2 receptor antagonist acts outside the blood-brain barrier (BBB) on the area postrema in the brain stem, which is a chemoreceptor trigger zone, causing antiemetic action. It also acts on the gastrointestinal tract where it functions as a prokinetic drug [2]. In India, domperidone is easily available as a combination drug with proton pump inhibitors (PPIs) for the management of dyspepsia and gastroesophageal reflux disease. In the United States, the Food and Drug Administration (FDA) has approved it in finite refractory gastrointestinal cases only [2].

Domperidone-producing extrapyramidal symptoms (EPS) are scarce as it does not traverse through the BBB in conventional circumstances [3] unlike its antiemetic partner metoclopramide [4] and structurally analogous drug, haloperidol, which does [5]. There are few other antipsychotic dopamine antagonist drugs where EPS is reasonably frequent in form of cranial dystonia (blepharospasm, oromandibular or lingual dystonia), oculogyric crisis, cervical spasmodic torticollis, spasmodic laryngeal dystonia, limb dystonia, writer’s cramp, akathisia, tardive dyskinesia and parkinsonism [5]. We present a rare case of domperidone-induced acute dystonia presented within 72 hours after consuming two doses of commonly available oral combination drug of PPIs with domperidone suggested for dyspepsia in a young adult.

## Case presentation

A 28-year-young male did an online telemedicine consultation for allergic rhinitis during the current coronavirus disease 2019 (COVID-19) pandemic. He was prescribed a nasal drop containing a combination of azelastine 140 mcg (a second-generation antihistaminic) and fluticasone 50 mcg (a topical steroid) to apply once daily for one week along with an oral anti-allergic combination tablet of levocetirizine (5 mg) and montelukast (10 mg) for five days. He has no co-morbidities but claims using the same nasal drop on and off for the last three years for allergic rhinitis. Current nasal symptoms improved after few days but he developed dyspepsia to which he started consuming a combination capsule orally containing PPI, rabeprazole (20 mg), and domperidone (30 mg) as this drug is easily obtainable over the counter in India.

After taking this new compound for around two days, the patient’s kin observed that occasionally while talking, the right lower half of his face would appear deviated and he had a sustained contraction to the right side for a prolonged time irrespective of body posture. There is no trouble while chewing, swallowing, or speaking; no noticeable alteration in voice or speech, and there was no body or limb weakness or jerks. The patient perceived a little sense of discomfort and tightness on the same side of face but no facial or retro auricular pain or swelling. There is no history of fall, trauma, resting tremors, gait disturbances or any recent fever. No family history of dystonia or any movement disorders. The patient assured that he is not on any antipsychotic, ayurvedic/ homeopathic, or any other traditional medication, is not alcoholic, and does not consume any illicit drugs. The patient had doubts regarding the relation of the symptoms to stress or emotions.

On examination, there was a slight head tilt to the right side, likely cervical dystonia, which the kindred also acknowledged. On observing the patient at rest and while speaking, there was an intermittent, non-rhythmic involuntary movement of right lower part of face prominently in perioral region in the form of facial twitches, with occasional deviation of the angle of mouth to right side amidst infrequent swallowing, most likely facial dystonia associated with laryngeal spasmodic dystonia. Cranial nerve examination was normal with no facial palsy. Meningeal signs were absent with no limb weakness. Reflexes and sensations were intact. As per kin, patient’s facial grimacing seems to be attenuated in the clinic. A non-rhythmic, irregular, transient dystonic movement of right index finger was spotted on outstretched hand. Basic blood investigation namely complete blood count, renal function with electrolytes, calcium, liver and thyroid function test along with urine routine examination were normal. Clinical impression was drug induced dystonia (DID) secondary to oral domperidone.

It was recommended to halt all current medications. The patient was promptly started on oral tetrabenazine (TBZ) at a dose of 12.5 mg once daily, gradually titrated to a twice-daily dose after five days, and continued for the next four weeks. In follow-up after a month, there was a complete resolution of dystonic features with no residual deficit. Patient did not opt for re-challenging domperidone for obvious reasons. Naranjo adverse drug reaction (ADR) probability score was 6, which infers probable relation with the drug in question.

## Discussion

PPIs combined with domperidone are freely available over the counter in India and the latter may trigger dystonia, as seen in our case. A comprehensive history is pivotal for precise clinical diagnosis as it staves off unneeded anti-epileptic drug prescription, series of blood investigations, imaging procedures, viz. computed tomography or magnetic resonance imaging scan, and invasive procedures, particularly lumbar puncture for cerebrospinal fluid analysis.

In an artificial environment, domperidone impressively binds to striatal dopamine receptors as the classical antipsychotics do [2], but multidrug resistance protein (MDR1, a p-glycoprotein efflux pump) expressed in the luminal surface [6] of BBB drives domperidone out from CNS contrary to its structurally related drug butyrophenone neuroleptic, haloperidol [7]. Only a handful of cases of domperidone-related dystonia and tardive dyskinesia have been published as yet [2,3,8], largely in the pediatric population ascribed to under-developed BBB or the elderly age group where the BBB permeability adjusts due to altered protein expression amidst neuronal modulation [3]. Table 1 enlists few drugs studied to cause EPS [3,4,9,10] and Table 2 illustrates the differentials that can be contemplated while evaluating a case of drug-induced movement disorders (DIMD) [3,5,11].

**Table 1:** Few drugs that cause EPS 5-HT2A: 5-hydroxy tryptamine2A; EPS: extrapyramidal symptoms

Sl. No.	Major category:	Different drug types:
1	Dopamine receptor blocker agents (DRBA)	Typical/first-generation antipsychotics: haloperidol, trifluoperazine, chlorpromazine
		Atypical/second-generation antipsychotics (attributes fewer EPS, which could be due to central serotonin-2A (5-HT2A) antagonism and D2 partial agonism): olanzapine, risperidone, fluphenazine, aripiprazole, amisulpride, levosulpiride, quetiapine
		Antiemetic: metoclopramide, prochlorperazine, domperidone
2	Non-dopamine receptor blocker agent (non-DRBA)	Selective serotonin reuptake inhibitors (SSRI)
		Antidepressants: amitriptyline, amoxapine
		Sedatives: midazolam
		Anticonvulsants: carbamazepine, phenytoin
		Antihistaminic: promethazine (first generation)
		Calcium channel blockers: flunarizine, verapamil
		Mood stabilizers: lithium

**Table 2:** Differentials for drug-induced movement disorders (DIMD) Recently Chouksey et al. [10] concluded in a study that tardive dystonia was the most common drug-induced movement disorder (DIMD) seen in DRBA and non-DRBA groups combined while postural tremor was the most frequent finding in the non-DRBA segment. Drug-induced parkinsonism (DIP) was prominent in the DRBA group DRBA: dopamine receptor blocker agent

Sl. No.	Medical conditions:
1	Encephalitis
2	Partial seizure
3	Parkinson’s disease
4	Wilson disease
5	Head trauma
6	Basal ganglia lesion (vascular, mass lesion)
7	Toxins
8	Tetany
9	Electrolyte imbalance
10	Vascular abnormalities
11	Hemifacial spasm
12	Ischemic brain injury during perinatal period (pediatric population)
13	Psychogenic (hysterical spasms)

The acquired dystonia in our case worsened with oral movements/talking suggesting that the central nervous system (CNS) might have chosen biased incorrect commands to perform a routine motor behavior/task. This could ramify in the overflow of muscular activity (perhaps a co-contraction of agonists and antagonists muscle group or may be a failed feedback controller) rather than activating a specific set of muscles to do that particular task in a synchronized scheme implying a perturbation of cortical sub-cortical neuronal network [12].

**Figure 1:**
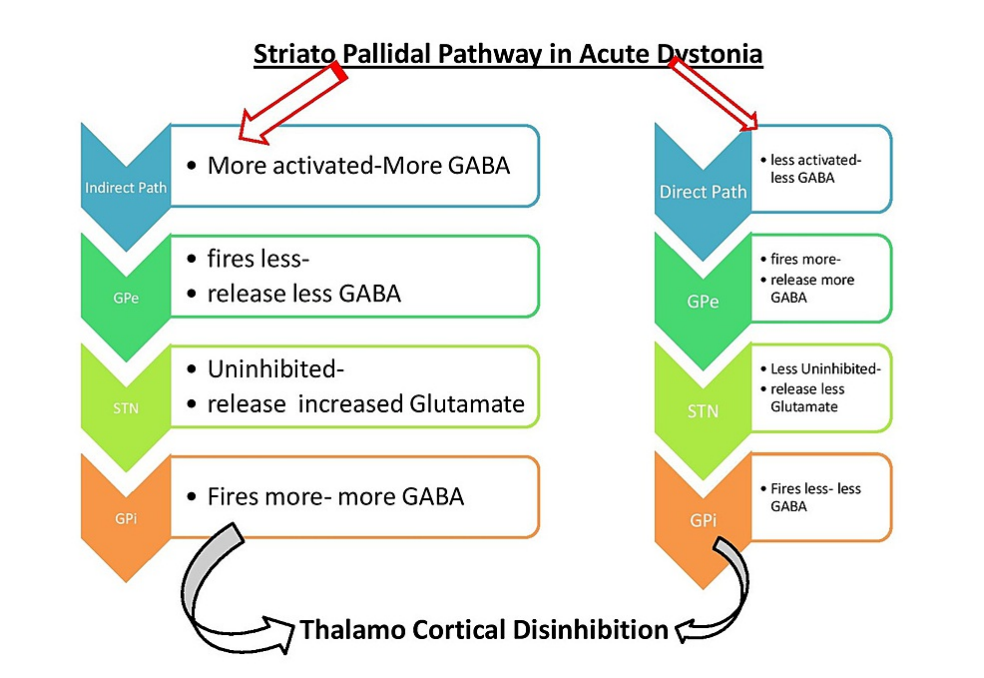
Striato-pallidal circuit is regulated by the amount of dopamine and other neurotransmitters along with its receptors. A simplified flowchart is illustrated here for drug-induced EPS. The direct pathway (direct-SPNs on right side) is inhibited and the indirect pathway (indirect-SPNs on left side) is activated. A combination of akinesia/severe bradykinesia and rigidity could be the end result of right and left side of flowchart respectively climaxing with dystonia. STN: subthalamic nuclei; GPi: globus pallidus internus; GPe: globus pallidus externa; SNr: substantia nigra pars reticularis; GABA: gamma amino butyric acid (neurotransmitter); EPS: extrapyramidal symptom; SPN: spiny projection neurons Source: Original illustration

**Figure 2:**
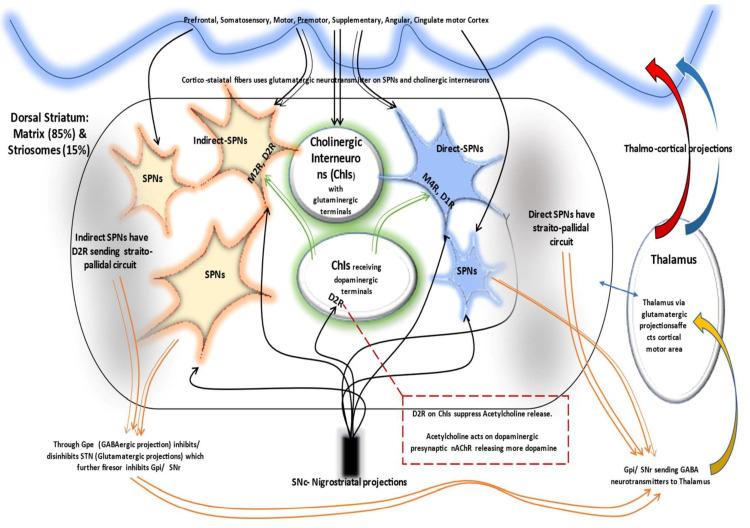
A simplified representation of different projections and interrelation of neuronal network in striatum. Cortical fibers project to direct SPNs, indirect SPNs, and cholinergic interneuron through glutamate neurotransmitter. In the direct path, the axons of SPNs converge on GPi relays back to cortex through thalamus. The indirect pathway maintains the antagonist portion of a voluntary movement via GPe through gabanergic projections. It finally converges on GPi through STN from where commences the common pathway to cortex via thalamus. Nigrostriatal projections have connections to direct, indirect, and cholinergic interneuron (large aspiny neurons) through a complex network of receptors and neurotransmitters (details not shown). The neuronal projections are illustrated in colorful curved lines. SPN: spiny projection neurons; GPi: globus pallidus interna; GPe: globus pallidus externa; M4R: mucarinic 4 receptors; STN: subthalamic nucleus; AChR: nicotine acetylcholine receptors; D1R: dopamine 1 receptor; D2R: dopamine 2 receptor; SPN: spiny projection neurons; SNc: substancia nigra compacta; SNr: substancia nigra reticularis; Chls: Cholinergic interneurons; GABA: gamma amino butyric acid (neurotransmitter) Source: Original illustration

Although the precise mechanism of drug-induced dystonia is still in dilemma [9,13], this could be explicated similar to the off-state of Parkinson’s disease (PD) (a hypokinetic state of PD), particularly off drug dystonic state or drug naïve dystonia cases of PD (implying non-dopamine state). The direct striatopallidal pathway goes inhibited and indirect one is activated relaying further to precipitate an outburst of neuronal excitability (high frequency discharge) of subthalamic nucleus (STN). This could reverberate into thalamocortical disinhibition; expressing as dystonic posture [12]. The situation could be visualized as a combination of increased indirect spiny projection neurons, iSPN activity (precipitating rigidity), and lessened direct spiny projection neurons (dSPN) activity (precipitating akinesia) [12]. This striato-pallidal activity is summarized in Figure 1 for easy understanding. A simplified representation of different projection along with neuronal network interrelation in striatum illustrating the direct and indirect circuit is displayed in Figure 2.

The supersensitivity theory is well known and is more appropriate for tardive dyskinesia instigated by anti psychotics. This theory asserts the chronic usage of drugs antagonizing post-synaptic dopamine D2 receptors (D2R) in striatum, sequel in upregulated post synaptic novel D2R density on spiny projection neurons (SPN) membranes [12]. The surplus stockpile of dopamine in the matrix adheres on these new unblocked D2R (as the routine D2R are blocked by the dopaminergic blockers, particularly antipsychotics), displaying a weak indirect striatopallidal trail compared to normal direct pathway. This weak indirect stimulation is sufficient to portray disinhibition of thalamocortical projection eliciting tardive dyskinesia/dystonia [12]. Sudden cessation of antipsychotics is not advisable as it would exponentiate the dyskinetic movements because of availability of the earlier blocked D2R to excess accumulated dopamine. In our case, sudden stoppage of domperidone did not lead to increase distress although this drug was served for less time span.

Cortico-striatal neuronal projections with post-synaptic glutamatergic receptors maintain striatum neuronal homeostasis and play a role in spike-timing-dependent neuro-synaptic plasticity via long term potentiation (LTP) and long-term depression (LTD). LTP and LTD can be interpreted as long-lasting induction and reduction of glutamate neurotransmitters. respectively, perhaps via upregulating or downregulating the NMDA and AMPA receptors [12]. Synaptic-plasticity is considered as an important neurophysiological phenomenon for memory forming from repetitive procedures in an attempt to endorse a skill and any unwanted neuroplastic alteration disrupting this synaptic input-output array could induce dystonia [9,12,13].

A thought on topical second-generation anti-histaminic as doubtful culprit drug

Another possibility in our case could be orofacial tardive dyskinesia due to prolong use of topical H2 receptor anti-histaminic azelastine, but there was no reproducibility of symptoms when it was reintroduced after a drug-free interval of one month for allergic rhinitis. Possibility of domperidone (a cytochrome p450 inhibitor) interacting with azelastine, raising its concentration was also contemplated.

First-generation H1 anti-histaminic crosses the BBB owing to its stunted molecular weight, lipophilicity, and low substrate recognition by P-glycoprotein [6] lamentably blunting cognition as an undesirable effect. Second-generation H1 anti-histaminic penetrates BBB in trivial amounts. It is worth mentioning that H1 receptor shares 45% homology with the muscarinic receptor, perhaps defending its usage as anticholinergic in treatment of dystonia and akathisia [6]. Rapid-acting intranasal topical second-generation H1 antihistamines such as azelastine (as in our case), epinastine, and others can have little systemic absorption [6], although rhinorrhea makes its absorption further questionable.

We found a case report of a long-term nasal decongestant user who apparently suffered blepharospasm and orofacial dystonia albeit obscure particulars [14] and recently, a case was identified where a single dose of an oral second-generation antihistaminic drug, cetirizine, triggered dystonia in a child [15]. These cases could advocate the doubtful role of second-generation anti-histaminic in DIMD.

Surprisingly, promethazine, a first-generation strong H1 receptor antihistaminic with moderate anticholinergic and anti-dopaminergic property can undo dystonia kindled by drugs; however, paradoxical cases have been noted where promethazine itself elicited acute dystonic activity [16]. In our case, antihistaminic nasal spray was reintroduced after a month without precipitating hyperkinetic disorder of any kind but a long-term follow-up is warranted to observe tardive dyskinesia.

In our case, the patient opted for oral medication management. Injectables were not used as the patient was not in distress and preferred to stay at home for safety concerns due to the COVID-19 pandemic. Tetrabenazine, a vesicular monoamine transporter 2 (VMAT2) inhibitor was used, which exhibited good tolerability and displayed excellent clinical improvement. This was approved by FDA for Huntington chorea [17,18] but off label, it has been used extensively in many hyperkinetic movement disorder treatments namely dystonia, dyskinesia, and tics [18]. The current modalities of treatment of dystonia are summarized and enlisted in Table 3 [3,9,11,19].

**Table 3:** Summary of a few known treatment options available for acute dystonia VMAT-2: vesicular monoamine transporter-2; GABA: gamma-aminobutyric acid

Sl. No.	Treatment modalities currently available for dystonia
1	Parenteral anticholinergic: benzatropine, diphenhydramine
2	Oral anticholinergic: trihexyphenidyl, start at a dose of 1 mg daily, can be increased by 1 mg every three to five days, goal dose 2 mg thrice daily, can be increased by 2 mg every week up to 30mg thrice daily.
3	First-generation antihistaminic with anti-cholinergic property: oral promethazine, oral or IV diphenhydramine (benadryl), oral chlorpheniramine
4	Tetrabenazine (TBZ): VMAT-2 inhibitor, start dose 12.5 mg once daily and can be titrated up every three to five days
5	GABA receptor agonist: baclofen, start dose 5 mg daily, increase by 5 mg/day every three to five days
6	Benzodiazepine: clonazepam, start at 0.5 mg
7	Dopaminergics: dopa responsive dystonia treated for childhood dystonia
8	Other agents: oral or IV lidocaine, alcohol, tizanidine
9	Botulinum toxin
10	Surgery: peripheral denervation, intrathecal baclofen, pallidotomy, thalamotomy, DBS (deep brain stimulation)

## Conclusions

Drug-induced dystonia is reasonably embarrassing for patients, an apprehensive situation for kindred, and regrettably often misdiagnosed as some psychiatric condition. Expeditious diagnosis of this entity is effective as it saves time, economical as it prevents unnecessary investigations, and ameliorates prognosis. In order to diagnose drug-induced dystonia at the earliest, a comprehensive history is pivotal.

PPIs combined with domperidone are freely available over the counter in India. Our case ignites alertness amongst practitioners in India over the judicious use of PPIs with domperidone, as the latter may trigger EPS as seen in our case. Such combination drugs can be prescribed if absolutely mandatory by the treating physician; otherwise, PPIs alone would be sufficient for dyspepsia management. Similar cases in the future would undoubtedly reinforce our findings.
